# Epidemiology of Histoplasmosis Outbreaks, United States, 1938–2013

**DOI:** 10.3201/eid2203.151117

**Published:** 2016-03

**Authors:** Kaitlin Benedict, Rajal K. Mody

**Affiliations:** Centers for Disease Control and Prevention, Atlanta, Georgia, USA

**Keywords:** histoplasmosis, Histoplasma, fungi, disease outbreaks, mycoses, United States, birds, bats, environment, epidemiology

## Abstract

Histoplasmosis has been described as the most common endemic mycosis in the United States. However, histoplasmosis is not nationally notifiable. Its presumed geographic distribution is largely derived from skin test surveys performed during the 1940s, and information about its local features comes primarily from outbreak investigations. We conducted a literature review to assess epidemiologic features of histoplasmosis outbreaks in the United States. During 1938–2013, a total of 105 outbreaks involving 2,850 cases were reported in 26 states and the territory of Puerto Rico. Common exposure settings were chicken coops and buildings or other structures undergoing renovation or demolition. Birds, bats, or their droppings were reported to be present in 77% of outbreak settings, and workplace exposures were reported in 41% of outbreaks. The continued occurrence of histoplasmosis outbreaks, particularly work-related ones involving known disturbance of bird or bat droppings, highlights the need to increase awareness of the disease.

## Abstract 2

Continued occurrence, particularly in work-related settings, highlights the need to increase awareness of this disease.

Histoplasmosis is caused by inhalation of the microconidia of *Histoplasma *spp. fungus, which are thermally dimorphic (i.e., environmental mold which converts to a yeast at 37°C). Infection can range from asymptomatic to life-threatening disease, depending on host status, inoculum size, and other factors (*1*). In the United States, histoplasmosis-endemic areas were established during the 1940s–1950s by using nationwide skin testing to evaluate histoplasmin sensitivity among young adults (*2*). The highest percentages of positive reactions (60%–90%) were noted in areas surrounding the Ohio and Mississippi River valleys; percentages of positive reactions decreased with increasing distance from these valleys (*2*). *Histoplasma* spp. grow particularly well in organic matter enriched with bird or bat droppings and likely exist in microfoci within and outside the broadly defined endemic regions (*3**–**5*). Environmental disruption of *Histoplasma* habitats is often a key factor associated with histoplasmosis outbreaks (*3**–**5*).

Although most infections are acquired sporadically, reports describing epidemiologic features of histoplasmosis in the United States usually involve investigations of localized outbreaks (*3*), which have not been comprehensively reviewed since the 1970s (*6*). We provide an update on the epidemiologic features of documented histoplasmosis outbreaks and identify potential opportunities to prevent them.

## Methods

During February 2015, we searched Medline, Embase, Scopus, CINAHL (Cumulative Index to Nursing and Allied Health Literature), ProQuest, and CAB (Centre for Agriculture and Biosciences) Abstracts without date or language restrictions and used combinations of the terms “histoplasmosis,” “*Histoplasma*,” “outbreak,” “cluster,” “epidemic,” and “United States.” Using the digital archive of scientific literature produced by the Centers for Disease Control and Prevention (CDC Stacks, http://stacks.cdc.gov/), we searched for reports published in Morbidity and Mortality Weekly Report before 1981. We reviewed references pertaining to outbreak investigations in all relevant articles. In addition, we searched abstracts from major infectious disease and epidemiology conferences and searched records from CDC’s Mycotic Diseases Branch for information about unpublished outbreaks and about details of investigations published as conference abstracts. We abstracted clinical and epidemiologic data of interest from reports of outbreaks that met inclusion criteria (Technical Appendix). The Cochran-Armitage test for trend was used to assess changes by decade.

An outbreak was defined as >2 cases of histoplasmosis associated with a common environmental source. Outbreaks were included if >1 case had laboratory evidence of histoplasmosis or if *Histoplasma* spp. were recovered from the common source. A case was defined as an illness clinically compatible with acute histoplasmosis, as determined by the authors of the original reports. Laboratory evidence of infection was defined as any of the following: positive culture or histopathology, presence of H or M immunoprecipitin bands on immunodiffusion, complement fixation titer >1:8, a positive *Histoplasma* antigen enzyme immunoassay result in urine or serum, or a positive serologic test, as stated in the report. We did not include clusters of histoplasmosis cases transmitted through organ transplantation or those involving infections acquired abroad. Prior outbreaks described anecdotally in published reports were not included unless the exact number of cases was stated.

Radiographic evidence of infection included pulmonary infiltrates, lesions, nodules, or cavitation; hilar or mediastinal lymphadenopathy; or unspecified findings indicating acute pulmonary histoplasmosis, as noted by the original authors. Outbreak onset was determined by the date on which the first patient became ill. We assessed reports for statements regarding average duration between suspected exposure and symptom onset and calculated the median. For outbreaks in which the incubation period was expressed as only a range, we calculated medians for the minimum and maximum number of days. Outbreak duration was defined as the interval between symptom onset of the first case and onset of the last case.

For reports not stating an exact number of patients hospitalized but mentioning a general proportion (e.g., “most patients were hospitalized”), we used a conservative estimate (i.e., 1 + half the number of patients). For reports not including number of deaths, we assumed that no deaths resulted if no patients were hospitalized or if all hospitalized patients recovered. We assumed that all cases occurred among adults >18 years of age if an outbreak occurred entirely among workers.

Setting was defined as the location where exposures occurred, such as a chicken coop, cave, or building. Farm settings not specifically associated with a chicken coop were classified as “farm.” We used “residential area” to classify an outdoor residential area that was not a farm and that had no exposures associated with a specific building or structure. Outbreaks in which persons were suspected to have been exposed throughout a city because of windborne dispersal of infectious material from a common source were classified as “citywide windborne.” When outdoor exposures resulted from a common activity, but no specific setting was described, we categorized the setting as “unspecified outdoor area.”

Specific activities suspected to have initiated the outbreak were not mutually exclusive and included soil disruption (e.g., digging or excavation); disruption of plant matter (e.g., trees, wood, leaves, or vegetable matter); demolition, construction, or renovation activities; caving; and known disturbance of large accumulations of bird or bat droppings (e.g., scraping droppings from a bridge or shoveling accumulations of droppings from a building’s roof). We also assessed each report for statements about the mere presence of birds, bats, or their droppings because some outbreaks were not related to obvious disturbance of droppings, but birds, bats, or droppings were described as present in the areas of suspected exposure. Outbreaks were categorized as work-related if at least 1 case occurred in a worker as a direct result of his or her occupational activities and if those activities were believed to have initiated the outbreak. Outbreaks were classified as having workplace exposures if some patients were exposed in their workplace but were not directly involved in the outbreak-initiating activities.

## Results

**Figure 1:**
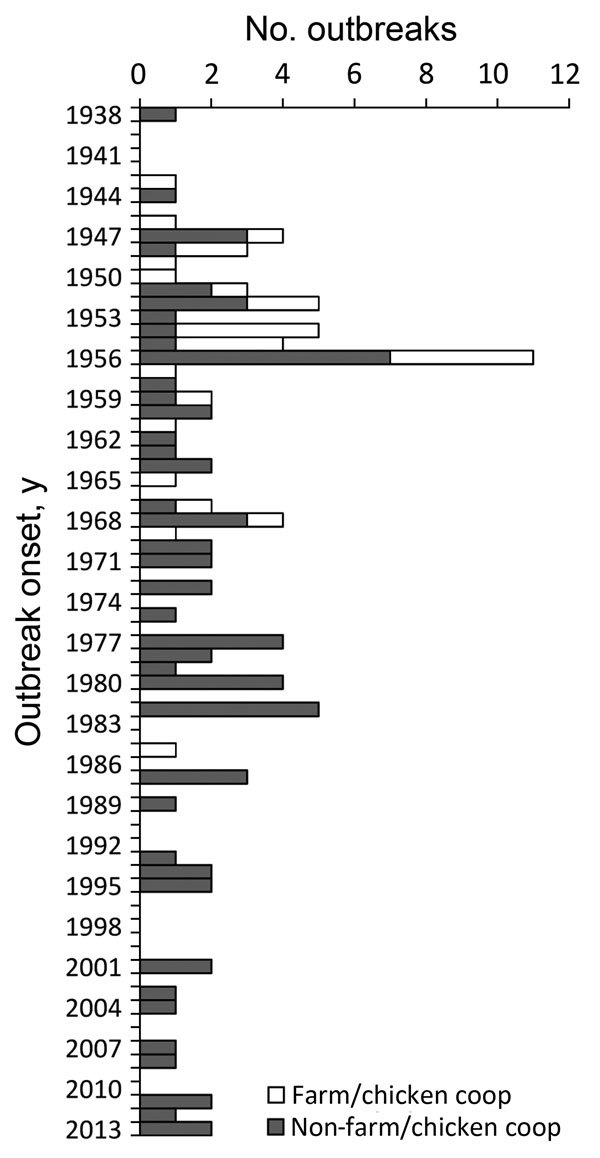
Number of histoplasmosis outbreaks by year of onset and setting, United States, 1938–2013 (N = 105).

**Figure 2:**
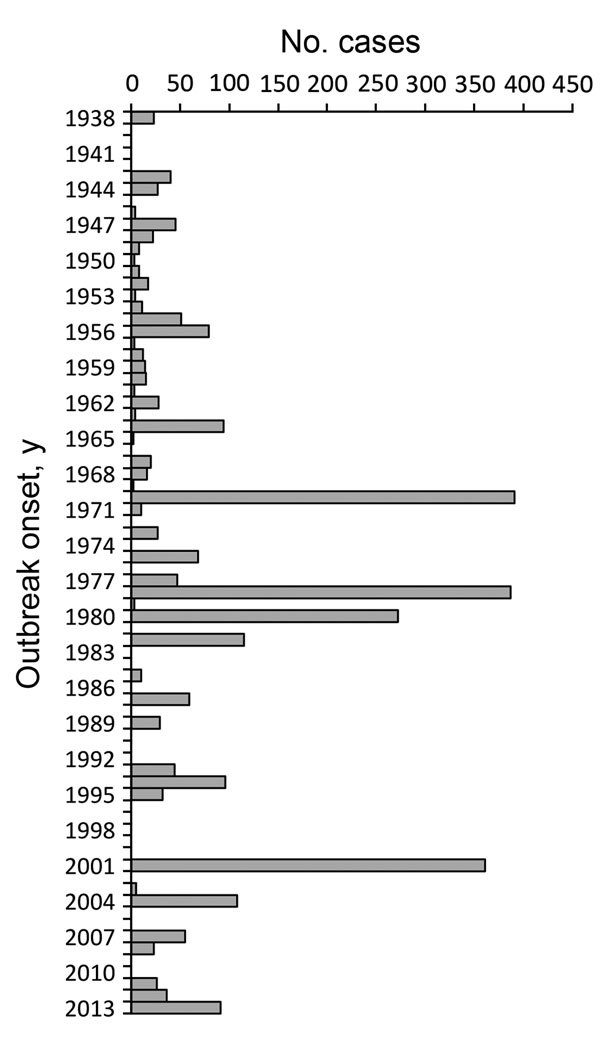
Number of outbreak-related cases of histoplasmosis by onset year, United States, 1938–2013 (N = 2,850).

This review includes 105 reported histoplasmosis outbreaks comprising 2,850 cases during 1938–2013 (Figures 1, 2). The range of outbreak size was 2–383 cases (mean 27; median 6). Seventeen (16%) outbreaks had 2 cases; 29 (28%) had 3–5; 18 (17%) had 6–9; 12 (11%) had 10–19; 10 (10%) had 20–29; and 19 (18%) had >30. All but 2 outbreaks had >1 case with laboratory evidence of histoplasmosis. Laboratory evidence was reported for 1,884 (66%) cases; 873 (31%) had no laboratory evidence; and 93 (3%) cases in 5 outbreaks had no information about percentage of cases with laboratory evidence.

**Figure 3:**
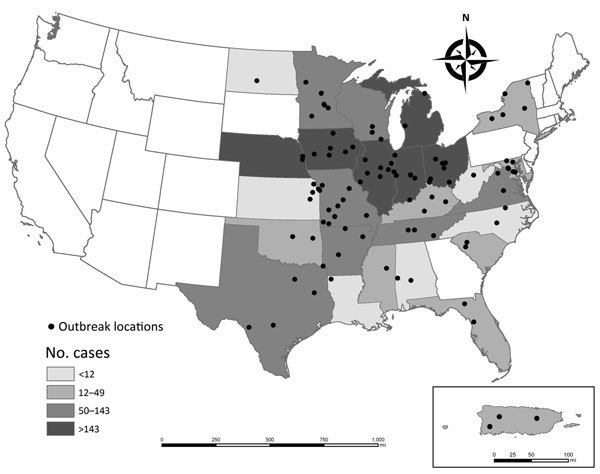
Locations of histoplasmosis outbreaks and number of outbreak-related cases, by state or territory (Puerto Rico, inset), United States, 1938–2013. City or county was not available for 1 outbreak in Ohio; 1 in Iowa; 2 in Tennessee; 1 in Missouri; and 1 in North Carolina. Points were placed in the center of the state for these outbreaks. Three of the 5 outbreaks in Puerto Rico occurred in the same cave system and appear as a single point on the map. Histoplasmosis is a reportable disease in Arkansas, Delaware, Illinois, Indiana, Kentucky, Michigan, Minnesota, Nebraska, Pennsylvania, and Wisconsin.

**Figure 4:**
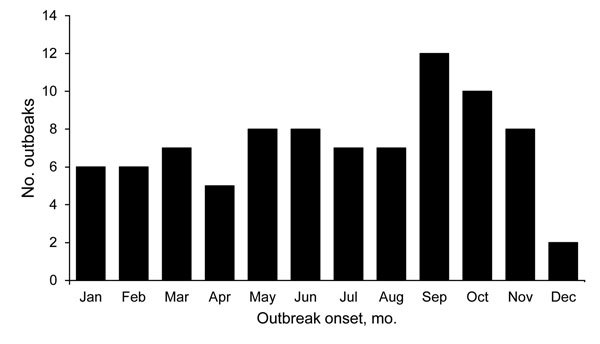
Number of histoplasmosis outbreaks by onset month (reported for 86 of 105 outbreaks), United States, 1938–2013.

Radiographic evidence of histoplasmosis was reported for 500 (81%) cases from 68 outbreaks. For 70 outbreaks that included 1,630 cases with complete data on patient age, 51% of cases occurred among children <18 years old. The preponderance of cases among children was driven by 2 large school-related outbreaks (*7**,**8*). If these 2 outbreaks are excluded, 82% of cases occurred among adults. For 53 outbreaks that included 1,318 cases with complete data about patient sex, 60% of cases occurred among males. Outbreaks were reported from 26 states and Puerto Rico (Figure 3). The following states had the most reported cases: Indiana, 790 (28%); Ohio, 415 (15%); Iowa, 213 (8%); Michigan, 182 (6%); Illinois, 155 (5%); Nebraska, 144 (5%); and Arkansas, 143 (5%). Onsets of most (72%) outbreaks occurred during May–November (Figure 4).

For 32 outbreaks with reported overall attack rates, the median attack rate was 63% (range 9%–100%). A median incubation period (median 10 days) was included in 5 reports; a mean (median 13 days) was included in 9 reports; and 25 reports stated a range. Median minimum and maximum incubation periods were 7 and 15 days, respectively. Median outbreak duration was 13 days (n = 51 outbreaks; range 1 d to >5 y).

### Treatment, Hospitalizations, and Outcomes

Information on antifungal therapy was reported for 34 outbreaks; 120 (6.7%) of 1,804 patients received antifungal treatment (Table 1). For reports of 62 outbreaks that included precise numbers of hospitalized patients, 265 (14.7%) of 1,801 patients were hospitalized. Inclusion of 2 large outbreaks with reported approximate numbers of hospitalized patients increased the minimum number of patients hospitalized to 610 (26.9%) of 2,269 (data not shown). The percentage of patients hospitalized generally decreased over the decades (p<0.0001), except for the 1970s, when a low number of patients were reported as hospitalized. For 72 outbreaks with death data, 25 (1.1%) of 2,232 patients died; as with hospitalizations, the percentage of patients who died decreased over time (p<0.0001) (Table 1).

**Table 1:** Antifungal drug treatments, hospitalizations, and deaths resulting from histoplasmosis outbreaks, by decade, United States, 1938–2013*

Decade	No. (%) patients		
	Treated	Hospitalized	Died
1938–1949	0	57 (64.8)	1 (1.7)
1950–1959	7 (21.9)	28 (37.3)	4 (3.3)
1960–1969	7 (6.5)	24 (48.0)	5 (2.9)
1970–1979	50 (5.5)	17 (3.7)	15 (1.8)
1980–1989	10 (4.5)	72 (27.5)	0 (0.0)
1990–1999	14 (20.6)	38 (22.1)	0 (0.0)
2000–2013	32 (6.8)	29 (4.1)	0 (0.0)
Total	120 (6.7)	265 (14.7)	25 (1.1)

*Percentages are of reported cases. Number and percent of cases from outbreaks were included when information was available.

### Settings and Activities

The most frequent settings for outbreaks were buildings (19 outbreaks), chicken coops (17 outbreaks), farms (12 outbreaks), and unspecified outdoor areas (11 outbreaks) (Table 2). Despite the frequency of outbreaks in these settings, outbreaks in these locations generally involved fewer cases than outbreaks in other settings. Citywide windborne outbreaks had the highest median number of cases (65, range 28–381); these cases were believed to have originated from environmental disturbances at a stream bank (2 outbreaks), a golf course (1 outbreak), and either an abandoned amusement park or a tennis complex (1 outbreak). Most (28/29) outbreaks associated with farms or chicken coops occurred during 1943–1969; the last occurred in 1985. More recent outbreaks (i.e., occurring during 1987–2013) were associated with various settings. The 9 cave-associated outbreaks represented some of the southernmost outbreak locations (Florida, Texas, and Puerto Rico); 4 of 5 outbreaks in Puerto Rico were associated with caves.

**Table 2:** Outbreaks and cases of histoplasmosis, by outbreak setting, United States, 1938–2013

Setting	No. outbreaks					No. cases*			
	Total	1938–1963	1964–1989	1989–2013		Total	Mean	Median	Range
Building	19	7	7	5		437	23	12	2–138
Chicken coop	17	14	3	0		105	6	4	2–40
Farm†	12	10	2	0		52	4	4	2–10
Outdoors, not specified	11	6	5	0		65	6	2	2–30
Cave‡	9	2	6	1		96	11	6	3–24
Residential area	7	4	2	1		74	11	8	3–26
Outdoor structure§	7	3	3	1		142	20	7	2–101
School or university	6	2	2	2		866	144	44	12–383
Other¶	5	2	0	3		195	39	27	6–108
Citywide windborne	4	1	3	0		538	135	65	28–381
Bamboo field	3	0	2	1		27	9	6	3–18
Prison	3	0	1	2		163	54	72	6–85
Campsite	2	0	1	1		90	45	45	36–54
All outbreaks	105	51	37	17		2,850	27	6	2–383

*Means and medians have been rounded to nearest whole number.  †Not specifically a chicken coop. ‡Four of 9 cave-associated outbreaks occurred in Puerto Rico. §Bridge (4 outbreaks), chapel (1 outbreak), and water tower (2 outbreaks). These outbreaks were work related except for the chapel-related outbreak. ¶Agricultural processing plant, storm cellar, park, landfill, and paper factory.

Disturbance of bird or bat dropping accumulations was described in 42 (40%) outbreaks; soil disruption in 34 (32%); plant matter disruption in 21 (20%); and demolition- or construction-related activities in 26 (25%). Details were insufficient to determine specific activities that may have precipitated 9 outbreaks. In 1 report, none of these activities was explicitly mentioned. Presence of bats (or bat droppings) was described in 24 (23%) outbreaks; presence of birds (or bird droppings) in 59 (56%); presence of either bird or bats in 81 (77%); and presence of birds and bats in 2 (2%). Reported birds included chickens (24 [41%] of 59 bird-related outbreaks); blackbirds, including starlings, grackles, and unspecified blackbirds (19 [32%]); pigeons (9 [15%]); and gulls (1 [2%]). Type of bird was not described in 8 (14%) bird-related outbreaks.

### Work-Related Outbreaks and Workplace Exposures

Thirty-five (33%) outbreaks were work-related; 26 occurred among workers only, and 9 affected workers and nonworkers. Fifteen (43%) work-related outbreaks took place at a building, 6 (17%) of which were outdoor structures: bridges (4 outbreaks) and water towers (2 outbreaks). Occupations involved in work-related outbreaks were primarily construction, demolition, or maintenance. Presence of birds, bats, or droppings were reported in 30 (86%) work-related outbreaks. For 8 outbreaks not classified as work related, some patients were exposed in their workplace but were not directly involved in outbreak-initiating activities. Altogether, cases acquired in the workplace were described in reports of 43 (41%) outbreaks. In 4 of those outbreaks, at least 1 public health investigator or laboratory worker became ill with histoplasmosis. 

### Other Features

Animals, primarily dogs, were infected in 5 outbreaks. In 6 outbreaks, >1 person was infected in a state other than the state of residence or other than the state where illness began. Of 72 outbreak investigations that used the histoplasmin skin test, 71 occurred before 1990. Environmental sampling was performed in 83 outbreak investigations; results were positive in 57 (69%). Environmental sampling was more commonly performed in outbreak investigations before 1990 than after (77/89 [87%] vs. 6/16 [38%]). Decontamination with formalin was described for 6 outbreaks (*7**,**9**–**13*).

## Discussion

During 1938–2013, a total of 2,850 cases of histoplasmosis resulted from 105 reported outbreaks in various settings in 26 US states and Puerto Rico. Outbreak locations were generally consistent with the known distribution of histoplasmosis; only a few outbreaks occurred in states believed to have a low level of endemicity (i.e., Florida, Minnesota, New York, North Dakota, and South Carolina [*2*]). The apparent decrease in the number of outbreaks over time may be largely because of the decline in reported farm- or chicken coop–associated outbreaks. However, the continued occurrence of histoplasmosis outbreaks highlights the need for increased awareness about ways to reduce exposures, particularly in the workplace and other settings where bird or bat droppings are present and environmental disruption occurs.

The association between histoplasmosis outbreaks and environmental disturbance, particularly in the presence of bird or bat droppings, is well recognized. We found 77% of outbreaks were reported to have evidence of bird or bat droppings; the actual percentage is likely higher, as our analysis was limited to data provided in published reports. The magnitude of environmental disturbance can range from minor, such as walking on contaminated ground or setting up tents (*14**,**15*), to large-scale, such as excavation or clearing foliage in a bird-roosting site (*13**,**16**,**17*). Among reports of outbreak investigations with sufficient information, only 1 outbreak was not described as associated with disturbance of bird or bat droppings, soil or plant matter disruption, or demolition or construction. This outbreak was suspected of being related to a load of coal that was dumped outside the windows of an Arkansas classroom, thus dispersing potentially contaminated coal dust; although chicken manure had been dumped on the school property during the previous year, the manure was last disturbed ≈6 months before the outbreak (*18*).

Many histoplasmosis outbreaks show the potential for cases to occur among persons who did not participate directly in the outbreak-initiating activities. Such cases often occurred in a workplace (*7**,**8**,**11**,**12**,**17**,**19**,**20*). More than 30% of outbreaks were work-related (i.e., with workers involved in outbreak-initiating activities), and ≈40% of outbreaks affected persons in their workplace (i.e., workers may or may not have been involved in the outbreak-initiating activities). For workers who disrupt contaminated soil or accumulations of bird or bat droppings, the National Institute for Occupational Safety and Health has developed guidance for workers and employers about ways to reduce exposures to *H. capsulatum*: excluding birds or bats from buildings; posting warnings and communicating health risks to workers; controlling dust during activities such as construction, demolition, and excavation in known endemic areas; properly disposing of potentially contaminated waste; and selecting and wearing appropriate personal protective equipment (PPE) (*21*). Several outbreak reports described cases among workers despite use of PPE; these cases confirm that PPE must be used correctly and consistently to be effective (*22**–**25*).

Our findings almost certainly underestimate the number of histoplasmosis outbreaks because fungal disease outbreaks are not nationally notifiable and many outbreaks likely go unpublished or unrecognized. Although diagnostic tests for histoplasmosis have improved during the past few decades, the infection can be challenging to diagnose, and outbreaks can be difficult to recognize. Even among outbreaks occurring since 1995, some patients had delayed diagnoses (*26*) or received unnecessary treatment for suspected bacterial infections (*8**,**26**,**27*). During a recent outbreak at a prison in Illinois, 42 inmates became ill within a 48-hour period (*28*); fever and headache were predominant symptoms, rather than respiratory symptoms, and a viral infection was initially suspected when 10 of 18 nasopharyngeal swabs tested positive for adenovirus but were negative with repeat testing (M.A. Arwady, unpub. data). This example highlights the nonspecific symptoms of acute pulmonary histoplasmosis (e.g., fever, cough, headache, fatigue, and chest pain), which can persist for weeks or months (*1**,**29**,**30*). Histoplasmosis can also be acquired outside the United States, so this illness should be considered for persons who have these symptoms and have recently traveled, especially to Central or South America. Outbreaks of internationally acquired histoplasmosis among travelers are known to occur, but those reports were outside the scope of this article.

In our analysis, the percentage (66%) of symptomatic patients with positive laboratory results likely underrepresents the true percentage of patients who would test positive because only a subset of ill persons were selected for or received laboratory testing during some outbreak investigations (*7**,**22*). Although we were unable to evaluate the type of diagnostic tests on an individual level, serologic tests are the most commonly used diagnostic method for acute pulmonary histoplasmosis. Complement fixation and immunodiffusion tests for histoplasmosis are each ≈80% sensitive, but antibodies can take up to 6 weeks to develop (*1*). A small number of reports in this analysis included cases with low complement fixation titers (>1:8). Although titers in this range are weak diagnostic evidence on their own, compatible signs, symptoms, and shared exposures strengthen the suspicion for histoplasmosis. *Histoplasma* antigen detection tests were first developed in the mid-1980s and are also useful for detecting acute disease, particularly among persons with immunocompromising conditions, disseminated histoplasmosis, or intense exposures (*1**,**31**,**32*).

Several examples show the potential for large histoplasmosis outbreaks. The outbreak with the most reported symptomatic cases (n = 383) occurred in 1970, when a group of junior high students in Ohio raked and swept a central courtyard where birds had roosted (*7*). A similarly large (but unusually prolonged and severe) citywide windborne outbreak of 381 symptomatic cases occurred during September 1978–August 1979 in Indianapolis, Indiana, where 2 activities and settings were suspected outbreak sources: demolition of an amusement park and construction of a tennis stadium (*30*). A serosurvey performed as part of that investigation revealed a large number of presumably asymptomatic persons who had laboratory evidence of acute histoplasmosis; the authors of the report extrapolated that >100,000 persons were infected (*30*). 

Generally, an estimated <1% of persons infected with *Histoplasma *spp. develop symptoms, and infection likely results in at least partial protection from future infection; however, reactivation histoplasmosis can occur in immunosuppressed persons (*1*). Thus, the true public health effects of a histoplasmosis outbreak are challenging to quantify, in part because some exposed persons may develop serious disease years after the exposure when they later develop immunocompromising conditions.

Most mild-to-moderate histoplasmosis cases are self limited, but patients with more severe cases require antifungal treatment (*29*). The percentage of histoplasmosis outbreak patients treated in each decade likely reflects the development of antifungal drugs commonly used to treat histoplasmosis: amphotericin B (approved by the Food and Drug Administration in 1958) and itraconazole (approved in 1992). Accordingly, the observed declines in outbreak-associated hospitalizations and deaths (*16**,**30**,**33**–**37*) over time may partially result from the improvement and availability of histoplasmosis-related treatment and diagnostic testing. We estimate that 15%–27% of patients in outbreaks require hospitalization but that only ≈1% of acute cases are fatal; however, this percentage of fatalities may be underestimated because we assumed that no deaths occurred during outbreaks in which no patients were hospitalized. 

Alternatively, decreased numbers of hospitalizations and deaths could be associated with changes in exposure types experienced at different outbreak settings. The apparent absence of chicken coop–associated outbreaks after the 1960s may reflect a true outbreak reduction related to an application of knowledge obtained during outbreak investigations but could also indicate a bias toward reduced reporting of these smaller outbreaks over time. With increased popularity of backyard chicken flocks over the past decade (*38*), public health officials and healthcare providers should continue to be aware of the potential for histoplasmosis outbreaks and sporadic cases related to keeping chickens.

Although the small peak in reported outbreaks and outbreak-related cases during the late 1970s and early 1980s coincided with the start of the HIV epidemic, the temporal association does not appear as strong as that described between sporadic cases and the HIV epidemic. Similarly, unlike sporadic cases, numbers of outbreaks or outbreak-related cases did not decrease appreciably after introduction of antiretroviral therapy (*39*), suggesting that histoplasmosis outbreaks are influenced more by environmental factors than by host factors.

Historically, skin testing, environmental testing, and decontamination of environmental material with formalin often played central roles in histoplasmosis outbreak investigation and control but are currently of limited or no relevance. The histoplasmin skin test was frequently used as an epidemiologic tool in outbreak investigations to test patients (either during the outbreak or as part of clinical follow-up) or other exposed persons or to establish skin-test positivity rates within a specific geographic area or population. Nevertheless, the skin test was not a useful diagnostic test because of concern about cross-reactivity with other endemic mycoses, and the reagents have not been commercially available in the United States since 2000 (*1**,**21*). Similarly, environmental recovery of *H. capsulatum* from outbreak settings contributed substantially to knowledge of this species’ natural habitat and provided essential epidemiologic linkage between cases and exposure settings. However, the current utility of environmental testing for *H. capsulatum* in outbreak situations is less clear because traditional culture-based detection methods are resource intensive, and detection of the organism in the environment likely would not change public health recommendations for outbreak control. Molecular methods to detect *H. capsulatum* in environmental samples appear promising but are not yet widely used. As these technologies advance, they may serve as faster, less expensive methods to analyze environmental samples than culture-based methods (*21*). Use of formalin to decontaminate contaminated soil or other environmental material is no longer recommended (*21*) because this substance is a health hazard and use is impractical for large areas or settings such as caves (*40*).

Overall, outbreak-associated cases likely represent a small proportion of histoplasmosis cases (*39*). Histoplasmosis is reportable to public health authorities in 10 states, but passive surveillance almost certainly underestimates the true number of diagnosed cases in these areas. Histoplasmosis is often described as the most common endemic mycosis in the United States. This description is perhaps accurate on the basis of the potentially large number of asymptomatic infections suggested by the nationwide skin test surveys of the 1940s–1950s (*2*); however, current estimates of symptomatic cases and of the economic and public health effects caused by histoplasmosis are unavailable. Future work is needed to better define the true burden of both outbreak-associated and sporadic cases of histoplasmosis. Increased awareness among healthcare providers, public health and occupational safety professionals, and the public is also needed so that appropriate methods can be used to reduce exposures in known endemic areas during disruption of bird or bat droppings or other contaminated material.

**Technical Appendix.** References for reports of histoplasmosis outbreaks described in this study, organized by setting, United States, 1938–2013.
