# Supplementary material for: Epidemiology of Histoplasmosis Outbreaks, United States, 1938–2013
Source: Emerg Infect Dis. 2016 Mar;22(3):370–8. doi: 10.3201/eid2203.151117 (PMC4766901; doi:10.3201/eid2203.151117)
Supplement: Supplementary file 1 — Technical Appendix. References for reports of histoplasmosis outbreaks described in this study, organized by setting, United States, 1938–2013. [file 15-1117-Techapp-s1.pdf]

Article DOI: http://dx.doi.org/10.3201/eid2203.151117

Epidemiology of Histoplasmosis Outbreaks, United States, 1938–2013

Technical Appendix

References for Reported Histoplasmosis Outbreaks by Setting, United States, 1938–2013*

Building

Bartlett PC, Vonbehren LA, Tewari RP, Martin RJ, Eagleton L, Isaac MJ, et al. Bats in the belfry: an outbreak of histoplasmosis. Am J Public Health. 1982;72:1369–72. http://dx.doi.org/10.2105/AJPH.72.12.1369

Centers for Disease Control and Prevention. Histoplasmosis—Kentucky, 1995. MMWR Morb Mortal Wkly Rep. 1995;44:701–3.

Centers for Disease Control and Prevention. Epidemiological reports—histoplasmosis. MMWR Morb Mortal Wkly Rep. 1956;5:1.

Centers for Disease Control and Prevention. Epidemiological reports—histoplasmosis. MMWR Morb Mortal Wkly Rep. 1956;5:8.

Chick EW, Bauman DS, Lapp NL, Morgan WK. A combined field and laboratory epidemic of histoplasmosis. Isolation from bat feces in West Virginia. Am Rev Respir Dis. 1972;105:968–71.

Dean AG, Bates JH, Sorrels C, Sorrels T, Germany W, Ajello L, et al. An outbreak of histoplasmosis at an Arkansas courthouse, with five cases of probable reinfection. Am J Epidemiol. 1978;108:36–46.

Fournier M, Quinlisk P, Garvey A. Histoplasmosis infections associated with a demolition site—Iowa, 2008 [abstract]. Presented at: 58th Annual Epidemic Intelligence Service Conference; 2009 Apr 20–24; Atlanta, Georgia, USA. p. 56–57 [cited 2015 Mar 16]. http://www.cdc.gov/eis/downloads/2009.eis.conference.pdf

Gordon MA, Ziment I. Epidemic of acute Histoplasmosis in western New York State. N Y State J Med. 1967;67:235–43.

Grant YT, Harlacher V, Austin C. Histoplasmosis infection among temporary laborers—Illinois, August–September 2011 [abstract]. Presented at: 61st Annual Epidemic Intelligence Service Conference; 2012 Apr 16–20; Atlanta, Georgia, USA. p. 63 [cited 2015 Mar 16]. http://www.cdc.gov/eis/downloads/2012.eis.conference.pdf

Grayston JT, Furcolow ML. The occurrence of histoplasmosis in epidemics; epidemiological studies. Am J Public Health Nations Health. 1953;43:665–76. http://dx.doi.org/10.2105/AJPH.43.6_Pt_1.665

Lehan PH, Furcolow ML. Epidemic histoplasmosis. J Chronic Dis. 1957;5:489–503. http://dx.doi.org/10.1016/0021-9681(57)90117-0

Parrott T Jr, Taylor G, Poston MA, Smith DT. An epidemic of histoplasmosis in Warrenton, North Carolina. South Med J. 1955;48:1147–50. http://dx.doi.org/10.1097/00007611-195511000-00002

Schoenberger CI, Weiner JH, Mayo FJ, Spellman J, Waltersdorff RG. Acute pulmonary histoplasmosis outbreak following home renovation. Md Med J. 1988;37:457–60.

Trivedi K, Miramontes R, Dufficy D, Park B, Soyemi K, Sharp K, et al. Histoplasmosis outbreak related to construction at a state facility—Iowa 2008 [abstract]. Presented at: Joint 48th Interscience Conference on Antimicrobial Agents and Chemotherapy and 46th Annual Meeting of the Infectious Diseases Society of America; 2008 Oct 25–28; Washington, DC, USA. Abstract M-725 [cited 2015 Mar 16]. https://idsa.confex.com/idsa/2008/webprogram/Paper27163.html

Waldman RJ, England AC, Tauxe R, Kline T, Weeks RJ, Ajello L, et al. A winter outbreak of acute histoplasmosis in Northern Michigan. Am J Epidemiol. 1983;117:68–75.

Younglove RM, Terry RM, Rose NJ, Martin RJ, Schnurrenberger PR. An outbreak of histoplasmosis in Illinois associated with starlings. IMJ Ill Med J. 1968;134:259–63.

Chicken Coop

Baum GL, Schwarz J, Floyd H. Epidemic histoplasmosis. Tuberculol Thorac Dis. 1959;18:30–3.

Beatty OA, Zwick LS, Paisley CG. Epidemic histoplasmosis in Ohio with source in Kentucky. Ohio State Med J. 1967;63:1470–2.

Grayston JT, Furcolow ML. The occurrence of histoplasmosis in epidemics; epidemiological studies. Am J Public Health Nations Health. 1953;43:665–76. http://dx.doi.org/10.2105/AJPH.43.6_Pt_1.665

Ibach MJ, Larsh HW, Furcolow ML. Epidemic histoplasmosis and airborne *Histoplasma capsulatum*. Proc Soc Exp Biol Med. 1954;85:72–4. http://dx.doi.org/10.3181/00379727-85-20789

Idstrom L, Rosenberg B. Primary Atypical Pneumonia. Bull U S Army Med Dep. 1944;81:88–92.

Lehan PH, Furcolow ML. Epidemic histoplasmosis. J Chronic Dis. 1957;5:489–503. http://dx.doi.org/10.1016/0021-9681(57)90117-0

Minor GR, Corey JH Jr. An epidemic of histoplasmosis in a family. Dis Chest. 1959;35:409–14. http://dx.doi.org/10.1378/chest.35.4.409

Rubin H, Lehan PH, Furcolow ML. Severe nonfatal histoplasmosis; report of a typical case, with comments on therapy. N Engl J Med. 1957;257:599–602. http://dx.doi.org/10.1056/NEJM195709262571304

Scalia SP. An outbreak of histoplasmosis in Baltimore County. Md State Med J. 1961;10:614–9.

Seward CW, Mohr JA, Rhoades ER. An outbreak of histoplasmosis in Oklahoma. Am Rev Respir Dis. 1970;102:950–8.

Welsh KS, Ildirim I, Furcolow ML. Case report: a familial microepidemic of histoplasmosis. J Ky Med Assoc. 1969;67:569–72.

Farm

Adriano SM, Schwarz J, Silverman FN. Epidemiologic studies in an outbreak of histoplasmosis. J Lab Clin Med. 1955;46:592–6.

Centers for Disease Control and Prevention. Follow-up tularemia—Indiana. MMWR Morb Mortal Wkly Rep. 1969;18:427.

Fuortes L, Hayes T. An outbreak of acute histoplasmosis in a family. Am Fam Physician. 1988;37:128–32.

Gordon MA, Greene CH, Elliott JC. A small outbreak of histoplasmosis. Albany (NY): New York State Department of Health; 1961.

Grayston JT, Furcolow ML. The occurrence of histoplasmosis in epidemics; epidemiological studies. Am J Public Health Nations Health. 1953;43:665–76. http://dx.doi.org/10.2105/AJPH.43.6_Pt_1.665

Grayston JT, Loosli CG, Alexander ER. The isolation of *Histoplasma capsulatum* from soil in an unused silo. Science. 1951;114:323–4. http://dx.doi.org/10.1126/science.114.2961.323

Lehan PH, Furcolow ML. Epidemic histoplasmosis. J Chronic Dis. 1957;5:489–503. http://dx.doi.org/10.1016/0021-9681(57)90117-0

Rutledge LH. An unusual type of pulmonary disease involving six members of a family. Minn Med. 1950;33:694–9.

Smith RT, Raetz SJ. Histoplasmosis; preliminary report of a family outbreak in Minnesota. Minn Med. 1955;38:531–7.

Outdoors, Not Specified

Centers for Disease Control and Prevention. Histoplasmosis—Texas. MMWR Morb Mortal Wkly Rep. 1972;21:231–6.

Cullen JH, Hazen E, Scholdager R. Two cases of histoplasmosis acquired in felling a decayed tree in the Mohawk Valley. N Y State J Med. 1956;56:3507–10.

Grayston JT, Furcolow ML. The occurrence of histoplasmosis in epidemics; epidemiological studies. Am J Public Health Nations Health. 1953;43:665–76. http://dx.doi.org/10.2105/AJPH.43.6_Pt_1.665

Kier JH, Campbell CC, Ajello L, Sutliff WD. Acute bronchopneumonic histoplasmosis following exposure to infected garden soil. J Am Med Assoc. 1954;155:1230–2. http://dx.doi.org/10.1001/jama.1954.73690320003010a

Lehan PH, Furcolow ML. Epidemic histoplasmosis. J Chronic Dis. 1957;5:489–503. http://dx.doi.org/10.1016/0021-9681(57)90117-0

Mack JK. The isolation of *Histoplasma capsulatum* from a hollow tree. J Pediatr. 1954;44:46–9. http://dx.doi.org/10.1016/S0022-3476(54)80090-4

Pladson TR, Stiles MA, Kuritsky JN. Pulmonary histoplasmosis. A possible risk in people who cut decayed wood. Chest. 1984;86:435–8. http://dx.doi.org/10.1378/chest.86.3.435

Ramírez J. Acute pulmonary histoplasmosis: newly recognized hazard of marijuana plant hunters. Am J Med. 1990;88:60N–2N.

Cave

Ashford DA, Hajjeh RA, Kelley MF, Kaufman L, Hutwagner L, McNeil MM. Outbreak of histoplasmosis among cavers attending the National Speleological Society Annual Convention, Texas, 1994. Am J Trop Med Hyg. 1999;60:899–903.

De Jesús LG, Ramos Morales F. Histoplasmosis in Puerto Rico. Three cases with infection from common source. Bol Asoc Med P R. 1968;60:501–8.

Díaz Martinez R. Epidemic of acute histoplasmasis in San German [in Spanish]. Bol Asoc Med P R. 1972;64:291–4.

Handzel S, Jessamine AG. Imported histoplasmosis from Puerto Rico. Can J Public Health. 1975;66:393–5.

Lottenberg R, Waldman RH, Ajello L, Hoff GL, Bigler W, Zellner SR. Pulmonary histoplasmosis associated with exploration of a bat cave. Am J Epidemiol. 1979;110:156–61.

McMurray DN, Russel LH. Contribution of bats to the maintenance of *Histoplasma capsulatum* in a cave microfocus. Am J Trop Med Hyg. 1982;31:527–31.

Sacks JJ, Ajello L, Crockett LK. An outbreak and review of cave-associated *histoplasmosis capsulati*. J Med Vet Mycol. 1986;24:313–25. http://dx.doi.org/10.1080/02681218680000471

Sifontes JE, Sotoviera ME, Torresdeblasini G. Histoplasmosis in Puerto Rico. Review and report of an outbreak occurring in Cuevas De Aguas Buenas [in Spanish]. Bol Asoc Med P R. 1964;56:445–52.

Washburn AM, Tuohy JH, Davis EL. Cave sickness, a new disease entity? Am J Public Health Nations Health. 1948;38:1521–6. http://dx.doi.org/10.2105/AJPH.38.11.1521

Residential Area

Byrd RB, Leavey R, Trunk G. The Chanute histoplasmosis epidemic. New variations of urban histoplasmosis. Chest. 1975;68:791–5. http://dx.doi.org/10.1378/chest.68.6.791

Campbell CC. A family outbreak of histoplasmosis. II. Epidemiologic studies. J Lab Clin Med. 1957;50:841–8.

Hosty TS, Ajello L, Wallace GD, Howell J, Moore J. A small outbreak of histoplasmosis. Am Rev Tuberc. 1958;78:576–82.

Perkins RL, Saslaw S, Ockner SA. Migration histoplasmosis. Ann Intern Med. 1962;57:363–72. http://dx.doi.org/10.7326/0003-4819-57-3-363

Smith R. Personal communication. March 2015.

Ward JI, Weeks M, Allen D, Hutcheson RH Jr, Anderson R, Fraser DW, et al. Acute histoplasmosis: clinical, epidemiologic and serologic findings of an outbreak associated with exposure to a fallen tree. Am J Med. 1979;66:587–95. http://dx.doi.org/10.1016/0002-9343(79)91168-9

Wilcox KR Jr, Waisbren BA, Martin J. The Walworth, Wisconsin, epidemic of histoplasmosis. Ann Intern Med. 1958;49:388–418. http://dx.doi.org/10.7326/0003-4819-49-2-388

School or University

Brodsky AL, Gregg MB, Loewenstein MS, Kaufman L, Mallison GF. Outbreak of histoplasmosis associated with the 1970 earth day activities. Am J Med. 1973;54:333–42. http://dx.doi.org/10.1016/0002-9343(73)90028-4

Chamany S, Mirza SA, Fleming JW, Howell JF, Lenhart SW, Mortimer VD, et al. A large histoplasmosis outbreak among high school students in Indiana, 2001. Pediatr Infect Dis J. 2004;23:909–14. http://dx.doi.org/10.1097/01.inf.0000141738.60845.da

Chin TD, Ney PE, Saltzman BN, Paxton GB, Rakich JH, Ware M, et al. An epidemic of histoplasmosis among school children in Arkansas. South Med J. 1956;49:785–92. http://dx.doi.org/10.1097/00007611-195608000-00001

Hagstrom RM. Epidemiologic studies by county health departments. Miss Doct. 1959;37:141–5.**PMID: 14398830**

Luby JP, Southern PM Jr, Haley CE, Vahle KL, Munford RS, Haley RW. Recurrent exposure to *Histoplasma capsulatum* in modern air-conditioned buildings. Clin Infect Dis. 2005;41:170–6. http://dx.doi.org/10.1086/430907

Schlech WF III, Wheat LJ, Ho JL, French ML, Weeks RJ, Kohler RB, et al. Recurrent urban histoplasmosis, Indianapolis, Indiana, 1980–1981. Am J Epidemiol. 1983;118:301–12.

Outdoor Structure

Davies SF. Serodiagnosis of histoplasmosis. Semin Respir Infect. 1986;1:9–15.

Centers for Disease Control and Prevention. Histoplasmosis—Lexington, Kentucky. MMWR Morb Mortal Wkly Rep. 1961;10:2–8.

Englert E Jr, Phillips AW. Acute diffuse pulmonary granulomatosis in bridge workers. Am J Med. 1953;15:733–40. http://dx.doi.org/10.1016/0002-9343(53)90161-2

Feldman HA, Sabin A. Pneumonitis of unknown etiology in a group of men exposed to pigeon excreta. J Clin Invest. 1948;27:533.

Huhn GD, Austin C, Carr M, Heyer D, Boudreau P, Gilbert G, et al. Two outbreaks of occupationally acquired histoplasmosis: more than workers at risk. Environ Health Perspect. 2005;113:585–9. http://dx.doi.org/10.1289/ehp.7484

Jones TF, Swinger GL, Craig AS, McNeil MM, Kaufman L, Schaffner W. Acute pulmonary histoplasmosis in bridge workers: a persistent problem. Am J Med. 1999;106:480–2. http://dx.doi.org/10.1016/S0002-9343(99)00044-3

Sorley DL, Levin ML, Warren JW, Flynn JPG, Gerstenblith J. Bat-associated histoplasmosis in Maryland bridge workers. Am J Med. 1979;67:623–6. http://dx.doi.org/10.1016/0002-9343(79)90244-4

Other

Cain JC, Devins EJ, Downing JE. An unusual pulmonary disease. Arch Intern Med (Chic). 1947;79:626–41. http://dx.doi.org/10.1001/archinte.1947.00220120056005

Centers for Disease Control and Prevention. Outbreak of histoplasmosis among industrial plant workers—Nebraska, 2004. MMWR Morb Mortal Wkly Rep. 2004;53:1020–2.

Furcolow ML, Tosh FE, Larsh HW, Lynch HJ Jr, Shaw G. The emerging pattern of urban histoplasmosis. Studies on an epidemic in Mexico, Missouri. N Engl J Med. 1961;264:1226–30. http://dx.doi.org/10.1056/NEJM196106152642402

Huhn GD, Austin C, Carr M, Heyer D, Boudreau P, Gilbert G, et al. Two outbreaks of occupationally acquired histoplasmosis: more than workers at risk. Environ Health Perspect. 2005;113:585–9. http://dx.doi.org/10.1289/ehp.7484

Stobierski MG, James Hospedales C, Hall WN, Robinson-Dunn B, Hoch D, Sheill DA. Outbreak of histoplasmosis among employees in a paper factory—Michigan, 1993. J Clin Microbiol. 1996;34:1220–3.

Citywide Windborne

D'Alessio DJ, Heeren RH, Hendricks SL, Ogilvie P, Furcolow ML. A starling roost as the source of urban epidemic histoplasmosis in an area of low incidence. Am Rev Respir Dis. 1965;92:725–31.

Sellers TF Jr, Price WN Jr, Newberry WM Jr. An epidemic of erythema multiforme and erythema nodosum caused by histoplasmosis. Ann Intern Med. 1965;62:1244–62. http://dx.doi.org/10.7326/0003-4819-62-6-1244

Tosh FE, Doto IL, D'Alessio DJ, Medeiros AA, Hendricks SL, Chin TD. The second of two epidemics of histoplasmosis resulting from work on the same starling roost. Am Rev Respir Dis. 1966;94:406–13.

Wheat LJ, Slama TG, Eitzen HE, Kohler RB, French ML, Biesecker JL. A large urban outbreak of histoplasmosis: clinical features. Ann Intern Med. 1981;94:331–7. http://dx.doi.org/10.7326/0003-4819-94-3-331

Bamboo Field

Centers for Disease Control and Prevention. Histoplasmosis—Northern Louisiana. MMWR Morb Mortal Wkly Rep. 1977;26:375.

Haselow DT, Safi H, Holcomb D, Smith N, Wagner KD, Bolden BB, et al. Histoplasmosis associated with a bamboo bonfire—Arkansas, October 2011. MMWR Morb Mortal Wkly Rep. 2014;63:165–8.

Sollod N. Acute fulminating disseminated histoplasmosis. Report of an unusual outbreak. J S C Med Assoc. 1971;67:231–4.

Storch G, Burford JG, George RB, Kaufman L, Ajello L. Acute histoplasmosis. Description of an outbreak in northern Louisiana. Chest. 1980;77:38–42. http://dx.doi.org/10.1378/chest.77.1.38

Prison

Arwady M, Vallabhaneni S, Tsai V, Smith R, Park B, Conover C. Febrile illness at a state correctional facility—Illinois, 2013 [abstract]. Presented at: IDWeek 2014; 2014 Oct 7–14; Philadelphia, Pennsylvania, USA. Abstract 1457 [cited 2015 Mar 16]. https://idsa.confex.com/idsa/2014/webprogram/Paper46191.html

Hajjeh RWD, Kaufman L, Padhye A, McNeil M. An outbreak of acute histoplasmosis in a correctional center in Virginia [abstract]. Presented at: 35th Interscience Conference on Antimicrobial Agents and Chemotherapy; 1995 Sep 17–20; San Francisco, California, USA. Abstract K201.

Morse DL, Gordon MA, Matte T, Eadie G. An outbreak of histoplasmosis in a prison. Am J Epidemiol. 1985;122:253–61.

Campsite

Gustafson TL, Kaufman L, Weeks R, Ajello L, Hutcheson RH, Wiener SL, et al. Outbreak of acute pulmonary histoplasmosis in members of a wagon train. Am J Med. 1981;71:759–65. http://dx.doi.org/10.1016/0002-9343(81)90361-2

Centers for Disease Control and Prevention. Histoplasmosis outbreak among day camp attendees—Nebraska, June 2012. MMWR Morb Mortal Wkly Rep. 2012;61:747–8.

*****References may be repeated for different settings
